# SEMA^3^: A free smartphone platform for daily life surveys

**DOI:** 10.3758/s13428-024-02445-w

**Published:** 2024-06-24

**Authors:** Sarah T. O’Brien, Nerisa Dozo, Jordan D. X. Hinton, Ella K. Moeck, Rio Susanto, Glenn T. Jayaputera, Richard O. Sinnott, Duy Vu, Mario Alvarez-Jimenez, John Gleeson, Peter Koval

**Affiliations:** 1 Melbourne School of Psychological Sciences, University of Melbourne, Melbourne, Australia; 2 School of Behavioural and Health Sciences, Australian Catholic University, Melbourne, Australia; 3 Australian Research Centre in Sex, and Society, La Trobe University, Melbourne, Australia; 4 School of Psychology, The University of Adelaide, Adelaide, Australia; 5 Melbourne eResearch Group, School of Computing and Information Systems, University of Melbourne, Melbourne, Australia; 6 Orygen, Melbourne, Australia; 7 Centre for Youth Mental Health, The University of Melbourne, Melbourne, Australia; 8 Healthy Brain and Mind Research Centre, The School of Behavioural and Health Sciences, Australian Catholic University, Melbourne, Australia

**Keywords:** Daily life, Intensive longitudinal methods, Ambulatory assessment, Smartphone surveys, Experience sampling method (ESM), Ecological momentary assessment (EMA), Daily diary method

## Abstract

Traditionally, behavioral, social, and health science researchers have relied on global/retrospective survey methods administered cross-sectionally (i.e., on a single occasion) or longitudinally (i.e., on several occasions separated by weeks, months, or years). More recently, social and health scientists have added *daily life survey methods* (also known as *intensive longitudinal methods* or *ambulatory assessment*) to their toolkit. These methods (e.g., daily diaries, experience sampling, ecological momentary assessment) involve dense repeated assessments in everyday settings. To facilitate research using daily life survey methods, we present SEMA^3^ (http://www.SEMA3.com), a platform for designing and administering intensive longitudinal daily life surveys via Android and iOS smartphones. SEMA^3^ fills an important gap by providing researchers with a free, intuitive, and flexible platform with basic and advanced functionality. In this article, we describe SEMA^3^’s development history and system architecture, provide an overview of how to design a study using SEMA^3^ and outline its key features, and discuss the platform’s limitations and propose directions for future development of SEMA^3^.

## Introduction

Psychology has a long tradition of conducting experimental laboratory research, and an almost equally long history of debating the (ecological) validity of lab-based findings (Black, 1955; Campbell, 1957; Diener et al., 2022; Mitchell, 2012; Mook, 1983; Schmuckler, 2001). It is not altogether surprising that people’s feelings, thoughts, and behavior can differ dramatically between artificial lab contexts and the complex environments they encounter in everyday life (Bolger et al., 2003; Trull & Ebner-Priemer, 2014; Wilhelm & Grossman, 2010).^1^ Moreover, some aspects of human psychology cannot be ethically or practically studied using experiments (Diener et al., 2022). Self-report survey methods are a popular alternative to experiments, which allow scientists to study a wide range of psychological processes outside the lab. Traditional self-report surveys ask respondents to summarize their psychological experience, behavior, or environment over relatively long periods of time (i.e., typically a week or longer). These methods are useful for assessing people’s memories and/or beliefs, but they cannot directly tap into momentary experience without bias (Schwarz, 2012). To capture “life as it is lived” (Bolger et al., 2003) requires naturalistic methods that assess momentary experience and behavior as they unfold, in vivo.

Fortunately, recent technological changes – especially the widespread adoption of smartphones – have made it much easier to study humans in their natural habitats (Harari et al., 2016; Miller, 2012). Three-quarters of adults in wealthy countries and almost half of all adults in emerging economies own a smartphone (Taylor & Silver, 2019). Furthermore, people use their smartphones frequently and consistently throughout the day (Andrews et al., 2015). For instance, a recent study using eye-level cameras to record smartphone use in daily life found that participants spent an average of 1 out of every 5 min on their smartphone (Heitmayer & Lahlou, 2021). The increasing ubiquity of smartphone use in everyday life underscores the usefulness of smartphone-based methods for studying human psychology “in the wild.” To this end, we introduce SEMA^3^, a free, flexible, and user-friendly platform for collecting daily life survey data available on Android and iOS smartphones.

In what follows, we begin by introducing *daily life survey methods*, including a brief history of the development and unique strengths of these methods. Next, we provide an overview of smartphone-based daily life survey methods and then we introduce the system architecture and key features of SEMA^3^. We then outline the key steps required to design a SEMA^3^ study before providing an overview of limitations and future directions of the platform.

### Daily life survey methods

Daily life survey methods can be distinguished from *traditional survey methods*, administered either cross-sectionally (i.e., at a single occasion) or longitudinally (i.e., at a handful of occasions, typically separated by months or years). Traditional survey methods require respondents to provide long-term retrospective (e.g., “over the past month/year”) or global (e.g., “in general”) self-reports, and therefore capture relatively stable, semantic knowledge or beliefs. In contrast, daily life surveys comprise repeated measurements of momentary (e.g., “right now”) or short-term retrospective (e.g., “today” or “since the last survey”) reports, which draw on current experience or episodic memory of recent experiences, respectively (Conner & Barrett, 2012; Robinson & Clore, 2002).

Some of the earliest applications of daily life survey methods were by psychologists, who have a long-standing interest in measuring the dynamics of people’s thoughts, feelings, and behaviors. For example, Flügle’s (1925) study of the daily emotional experiences of nine adults who reported their subjective feelings roughly once per hour for 30 days, and McCance et al.’s (1937) study of 167 women who reported on their menstruation symptoms, and feelings of sexual desire, depression, and irritability each day over 4–6 months. Such early examples of daily life research are rare, likely because this approach relied on participants’ ability and motivation to remember to complete pencil-and-paper surveys each day. The feasibility of daily life methods increased substantially with the development of electronic devices (e.g., wristwatches, pagers) that could be programmed to prompt participants to complete paper-and-pencil surveys, and later devices (e.g., personal digital assistants; PDAs) that could both prompt participants and record their survey responses (Wilhelm et al., 2012).

**Fig. 1:**
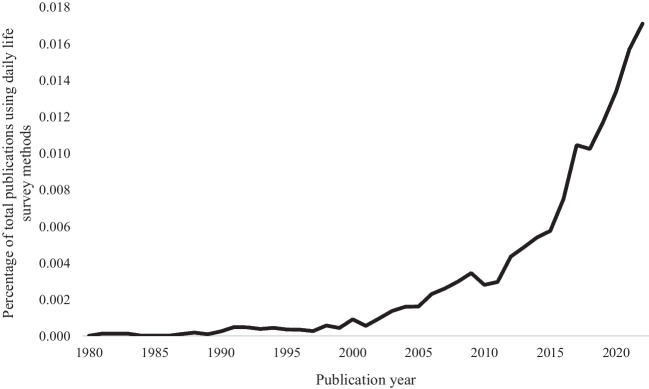
Total publications including daily life survey methods as a percentage of total publications across all fields from 1980–2022. *Note.* Publications including the terms “experience sampling”, “ecological momentary assessment”, “electronic diary” or “ambulatory assessment” in their title or abstract, from 1980 to 2022, as a percentage of total publications across all fields with no key words filtered, indexed by Dimensions https://www.dimensions.ai/. A percentage was used to indicate that these methods have increased in popularity above and beyond the increase in total scientific output. This plot is not cumulative.

Naturalistic, intensive repeated surveys have become increasingly common in the 21st century (see Fig. 1). Here, we collectively refer to this family of approaches as *daily life survey methods*, following Mehl and Conner (2012). These approaches include the *experience sampling method* (ESM; Csikszentmihalyi & Larson, 1987), *ecological momentary assessment* (EMA; Stone & Shiffman, 1994), and *diary methods* (Bolger et al., 2003). Other terms used for these and related approaches are *intensive longitudinal methods* (Bolger & Laurenceau, 2013) and *ambulatory assessment* (Trull & Ebner-Priemer, 2014). At their core, daily life survey methods involve frequent (i.e., typically at least once daily or more often) assessment of momentary (or very recent) experience, behavior, and/or context, as people go about their usual daily activities, typically over a relatively short time span (i.e., 1–4 weeks; Wrzus & Neubauer, 2023).

Several excellent sources already provide in-depth discussions of the unique strengths and limitations of daily life survey methods (e.g., Bolger et al., 2003; Hamaker, 2012; Hamaker & Wichers, 2017; Ram et al., 2017; Schwarz, 2012; Scollon et al., 2003; Shiffman et al., 2008; Trull & Ebner-Priemer, 2013, 2014). We therefore do not review these strengths in detail here but instead highlight three key points. First, daily life survey methods and traditional surveys do not necessarily produce converging results (e.g., Koval et al., 2023). Second, while some researchers have suggested that divergence between daily life and traditional surveys undermines the validity of one or both approaches, we see both as providing complementary information (Conner & Barrett, 2012; Finnigan & Vazire, 2018; Lucas et al., 2021). Third, given the unique characteristics and affordances of daily life survey methods, these methods are ideally suited to addressing research questions about short-term, within-person dynamic processes, and individual differences therein (e.g., Pauw et al., 2022; Van Reyn et al., 2023). For detailed examples of research questions to which daily life methods can be applied, as well as recommendations for how to analyze daily life data, we refer readers to Mehl and Conner’s (2012) *Handbook of Research Methods for Studying Daily Life*, as well as the more recent *Open Handbook of Experience Sampling Methodology*, edited by Myin-Germeys and Kuppens (2022). Furthermore, we present a list of recent publications reporting findings from daily life surveys collected using SEMA^3^ in Table 1.

**Table 1:** Examples of recent publications using data collected with SEMA

Field	Research topic	Publication details
Emotion & emotion regulation	Frequency and psychological effects of using smartphones for emotion regulation	Shi, Y., Koval, P., Kostakos, V., Goncalves, J., & Wadley, G. (2021). “Instant Happiness”: Smartphones as Tools for Everyday Emotion Regulation. *International Journal of Human-Computer Studies, 170.* 10.1016/j.ijhcs.2022.102958
	Interpersonal emotion regulation in daily life	Tran, A., Greenaway, K.H., Kostopoulos, J., O'Brien, S. T., & Kalokerinos, E. K. Mapping Interpersonal Emotion Regulation in Everyday Life. *Affective Science, 4,* 672–683 (2023). 10.1007/s42761-023-00223-z
	Influence of perceived social support on use and affective consequences of social emotion regulation strategies (social sharing and expressive suppression)	Pauw, L.S., Medland, H., Paling, S.J., Moeck, E. K., Greenaway, K. H., Kalokerinos, E. K., Hinton, J. D. X., Hollenstein, T., & Koval, P. Social Support Predicts Differential Use, but not Differential Effectiveness, of Expressive Suppression and Social Sharing in Daily Life. *Affective Science, 3, *641–652 (2022). 10.1007/s42761-022-00123-8
	Motivational strength in emotion regulation	Gutentag, T., Kalokerinos, K., Garrett, P., Millgram, Y., Sobel, R., & Tamir, M. (2021). Just Do It! Motivational Strength in Emotion Regulation (under review)
	Affective forecasting in daily life and relationship with well-being	Moeck, E., Grewal, K., Greenaway, K. H., Koval, P., & Kalokerinos, E. K. (2022). Everyday Affective Forecasting is Accurate, But Not Associated with Well-Being. 10.31234/osf.io/sr9vj (pre-print)
	Influence of social sharing on emotion differentiation in daily life	Sels, L., Erbas, Y., O'Brien, S. T., Verhofstadt, L., Clark, M. S., & Kalokerinos, E. K. (2022). To Share or Not to Share: Social Sharing Predicts Decreased Emotion Differentiation When Rumination is High. 10.31234/osf.io/y3cvu (under review)
Body image	Relationship between stress and body dissatisfaction and in daily life	Dang, A., Fuller-Tyszkiewicz, M., De La Harpe, S., Rozenblat, V., Giles, S., Kiropoulos, L. & Krug, I. (2021). Do women with differing levels of trait eating pathology experience daily stress and body dissatisfaction differently? *European Psychiatry, 64(S1),* S704-S705*.* 10.1192/j.eurpsy.2021.1866
	Influence of appearance-based comments, and social and performance-based evaluations on body dissatisfaction and disordered eating urges in daily life	Liu, S., Fuller-Tyszkiewicz, M., Eddy, S., Liu, X., Portingale, J., Giles, S., & Krug, I. (2022). The effects of appearance-based comments and non-appearance-based evaluations on body dissatisfaction and disordered eating urges: An Ecological Momentary Assessment Study. *Behavior Therapy, 53(5)*, 807–818. 10.1016/j.beth.2022.01.002
	Influence of women’s dating app use on body dissatisfaction, disordered eating urges, and negative mood in daily life	Portingale, J., Fuller-Tyszkiewicz, M., Liu, S., Eddy, S., Liu, X., Giles, S., & Krug, I. (2022). Love me Tinder: The effects of women’s lifetime dating app use on daily body dissatisfaction, disordered eating urges, and negative mood. *Body Image, 40*, 310–321. 10.1016/j.bodyim.2022.01.005
	Effects of food delivery app use, loneliness, and mood on body dissatisfaction and disordered eating urges in daily life	Portingale, J., Eddy, S., Fuller-Tyszkiewicz, M., Liu, S., Giles, S., & Krug, I. (2023). Tonight, I’m disordered eating: The effects of food delivery app use, loneliness, and mood on daily body dissatisfaction and disordered eating urges. *Appetite*, *180*. 10.1016/j.appet.2022.106310
Public health	Role of parent-child relationship quality on the impact of COVID-19 in the daily lives of adolescents	Janssens, J. J., Achterhof, R., Lafit, G., Bamps, E., Hagemann, N., Hiekkaranta, A. P., Hermans, K. S., Lecei, A., Myin‐Germeys, I., & Kirtley, O. J. (2021). The impact of Covid‐19 on adolescents’ daily lives: The role of parent–child relationship quality. *Journal of Research on Adolescence*, *31*(3), 623–644. 10.1111/jora.12657
	RCT of episodic future thinking and compassion exercises on public health guidelines noncompliance urges	van Baal, S., Verdejo-García, A., & Hohwy, J. (2021). Episodic future thinking and compassion reduce public health guideline noncompliance urges: A randomised controlled trial. 10.1101/2021.09.13.21263407 (pre-print)
	Development of an mHealth mental imagery-based intervention targeting reward sensitivity	Marciniak, M. A., Shanahan, L., Myin-Germeys, I., Veer, I., Yuen, K. S. L., Binder, H., Walter, H., Hermans, E., Kalisch, R., & Kleim, B. (2022). Imager – an mHealth mental imagery-based ecological momentary intervention targeting reward sensitivity: A randomized controlled trial. 10.31234/osf.io/jn5u4 (pre-print)
	COVID-19-related worries over time	Schulz, P. J., Andersson, E. M., Bizzotto, N., & Norberg, M. (2021). Using ecological momentary assessment to study the development of covid-19 worries in Sweden: Longitudinal Study. *Journal of Medical Internet Research*, *23*(11). 10.2196/26743
	Variability over time of emotions, physical complaints, and intention and self-efficacy towards physical activity in older adults	Maes, I., Mertens, L., Poppe, L., Crombez, G., Vetrovsky, T., & Van Dyck, D. (2022). The variability of emotions, physical complaints, intention, and self-efficacy: An ecological momentary assessment study in older adults. *PeerJ*, *10*. 10.7717/peerj.13234
	Impact of non-residential grandchild care on physical activity and sedentary behavior in people over 50 years	Vermote, M., Deliens, T., Deforche, B., & D’Hondt, E. (2021). The impact of non-residential grandchild care on physical activity and sedentary behavior in people aged 50 years and over: Study protocol of the healthy grandparenting project. *BMC Public Health*, *21*(1). 10.1186/s12889-020-10024-9
	Emotional functioning in COVID-19 lockdowns	Moeck, E. K., Freeman-Robinson, R., O'Brien, S. T., Woods, J. H., Grewal, K. K., Kostopoulos, J., Bagnara, L., Saling, Y. J., Greenaway, K. H., Koval, P., & Kalokerinos, E. K. (2023). Everyday emotional functioning in COVID-19 lockdowns. *Emotion.* 10.1037/emo0001226
	Social interaction dynamics in COVID-19 lockdowns	Tran, A., Bianchi, V., Moeck, E. K., Clarke, B., Moore, I., Burney, S. J. H., Koval, P., Kalokerinos, E. K., & Greenaway, K. H. (2023). Dynamics of Social Experiences in the Context of Extended Lockdown. *Social Psychological and Personality Science, 0*(0). 10.1177/19485506231176603
Clinical psychology	RCT of optimizing outcomes in psychotherapy for anxiety disorders protocol	Müller-Bardorff, M., Schulz, A., Paersch, C., Recher, D. A., Schlup, B., Seifritz, E., Kolassa, I.-T., Kleim, B., Kowatsch, T., Fisher, A. J., & Galatzer-Levy, I. (2022). Optimizing Outcomes in psychotherapy for anxiety disorders (OPTIMAX) protocol– a randomized controlled trial on efficacy and response prediction in a transdiagnostic psychotherapy treatment for anxiety disorders. 10.31234/osf.io/yezaj (pre-print)
	Relationship between daily social interactions and psychopathology in the context of COVID-19	Achterhof, R., Myin-Germeys, I., Bamps, E., Hagemann, N., Hermans, K. S., Hiekkaranta, A. P., Janssens, J., Lecei, A., Lafit, G., & Kirtley, O. J. (2021). Covid-19-related changes in adolescents’ daily-life social interactions and psychopathology symptoms. 10.31234/osf.io/5nfp2 (pre-print)
	Relationship between core-beliefs, bivalent fear of evaluation, and social anxiety symptoms	Cook, S. I., Felmingham, K. L., & Phillips, L. J. (2021). Relationships between core-beliefs, bivalent fear of evaluation, and social anxiety symptoms: A structural equation model (under review)
Memory	People’s accuracy in remembering where they were at a particular time in the recent past.	Laliberte, E., Yim, H., Stone, B., & Dennis, S. J. (2021). The Fallacy of an Airtight Alibi: Understanding Human Memory for “Where” Using Experience Sampling. *Psychological Science, 32(6)*, 944–951. 10.1177/0956797620980752
Self-concept	Development and validation of the Positive Evaluation Core Beliefs scale	Cook, S. I., Bryant, C., & Phillips, L. J. (2021). Development and validation of the Positive Evaluation Core Beliefs Scale (under review)
	Development of a momentary self-concept clarity scale	Ellison, W. D., Yun, J., Lupo, M. I., Lucas-Marinelli, A. K., Marshall, V. B., Matic, A. F., & Trahan, A. C. (2021). Development and initial validation of a scale to measure momentary self-concept clarity. *Self and Identity*, *8,* 995-1014. 10.1080/15298868.2021.2010796
Self-control	Influence of state impulsivity on urges (e.g., to snack, drink alcohol, gamble, etc.) and self-control in daily life	van Baal, S. T., Moskovsky, N., Hohwy, J., & Verdejo-García, A. (2022). State impulsivity amplifies urges without diminishing self-control. *Addictive Behaviors*, *133*. 10.1016/j.addbeh.2022.107381
	Temporal dynamics of mental imagery, craving and consumption of craved foods	Zorjan, S., & Schienle, A. (2022). Temporal dynamics of mental imagery, craving and consumption of craved foods: An experience sampling study. *Psychology & Health*, 1–17. 10.1080/08870446.2022.2033239
Stress	RCT of digital self-efficacy training in university students with self-reported elevated stress	Rohde, J., Marciniak, M. A., Henninger, M., Homan, S., Ries, A., Paersch, C., Friedman, O., Brown, A., & Kleim, B. (2022). Effects of a brief digital self-efficacy training in university students with self-reported elevated stress: A randomized controlled trial. 10.31234/osf.io/hkwm9 (pre-print)
Personality	Relationship between personality and attitudes, and daily pro-environmental behavior	Kesenheimer, J. S., & Greitemeyer, T. (2022). Going green is exhausting for dark personalities but beneficial for the light ones: An experience sampling study that examines the subjectivity of pro-environmental behavior. *Frontiers in Psychology*, *13*. 10.3389/fpsyg.2022.883704

## Smartphone-based daily life survey methods

As the ubiquity of smartphones has increased, so too have the benefits of using smartphone apps to collect daily life survey data. This allows researchers to reach large and diverse samples, and does not require participants to carry a dedicated research device that could alter the way they behave (Bailon et al., 2019). Dozens of commercial smartphone-based daily life survey platforms have been developed in recent years, with some offering limited free plans (e.g., m-Path, ExpiWell) and others requiring paid subscriptions (e.g., Metricwire, LifeData, ilumivu). The costs of such platforms mean that daily life survey methods remain inaccessible for many researchers. A few free platforms also exist, but these vary in their ease-of-use, with some requiring significant programming skills (e.g., ExperienceSampler, Thai & Page-Gould, 2018; formr, Arslan et al., 2020; PIEL Survey, Jessup et al., 2012). That being said, these platforms certainly have their merits and may be preferable to SEMA^3^ in certain cases. For example, for more complex studies involving question types or functions not available in SEMA^3^, it may be possible to create fully customized apps with other platforms (e.g., ExperienceSampler and formr), providing maximal flexibility. We believe the greatest benefit of SEMA^3^ over other available free platforms is the easy-to-use web-based researcher portal, which greatly simplifies study set-up and data monitoring. For a relatively recent comparison of daily life survey platforms, including SEMA^3^, we refer readers to Table 6.1 in Myin-Germeyz and Kuppens (2022).

Due to the sharp increase in daily life survey methods in recent years and their continued popularity, there is the space, and need, for multiple platforms to conduct this research. SEMA^3^ fills an important gap by providing researchers around the world with a free, highly intuitive, easy-to-use, and flexible platform to conduct research using daily life survey methods, thus helping to extend the reach of such methods beyond WEIRD (i.e., Western, educated, industrialized, rich, and democratic) researchers and samples (Rad et al., 2018).

### Introducing SEMA^3^

#### Development history

SEMA was originally designed in 2013–2014 with the aim of building a flexible smartphone-based EMA research platform, primarily for use in clinical intervention research. Following extensive pilot testing, SEMA was deployed in the Horyzons trial, a randomized controlled trial of an online psychosocial intervention for young people recovering from first-episode psychosis (Alvarez-Jimenez et al., 2021; Engel et al., 2024). Our experiences with the first version of SEMA in the Horyzons trial (including feedback from participants and research assistants) and in other smaller research projects led to a major redesign of the platform in 2015. The updated SEMA^2^ platform included several significant upgrades, including support for an offline data collection mode, improved data security, greater flexibility in survey and sampling-schedule design (e.g., basic formatting, randomization, conditional branching, survey versioning, participant-triggered surveys for event-contingent sampling), a new “demo survey” feature, and an improved data-monitoring dashboard. SEMA^2^ was active from 2015 to 2019, during which time it was used in dozens of research projects globally, including research on emotional experience and regulation (Grommisch et al., 2020; Haines et al., 2016; Koval et al., 2023; Medland et al., 2020), sexual objectification (Holland et al., 2017; Koval et al., 2019), and in clinical interventions (Gleeson et al., 2017; Gleeson et al., 2021; Weller et al., 2018). In 2019, the platform underwent further major upgrades and was re-released in its current version as SEMA^3^. In particular, SEMA^3^ addresses the need for (i) greater flexibility in survey and schedule design; (ii) new question types, (iii) personalized graphical feedback to participants; and (iv) improved, scalable back-end design and a unified codebase for both iOS and Android phones.

#### System architecture

**Fig. 2:**
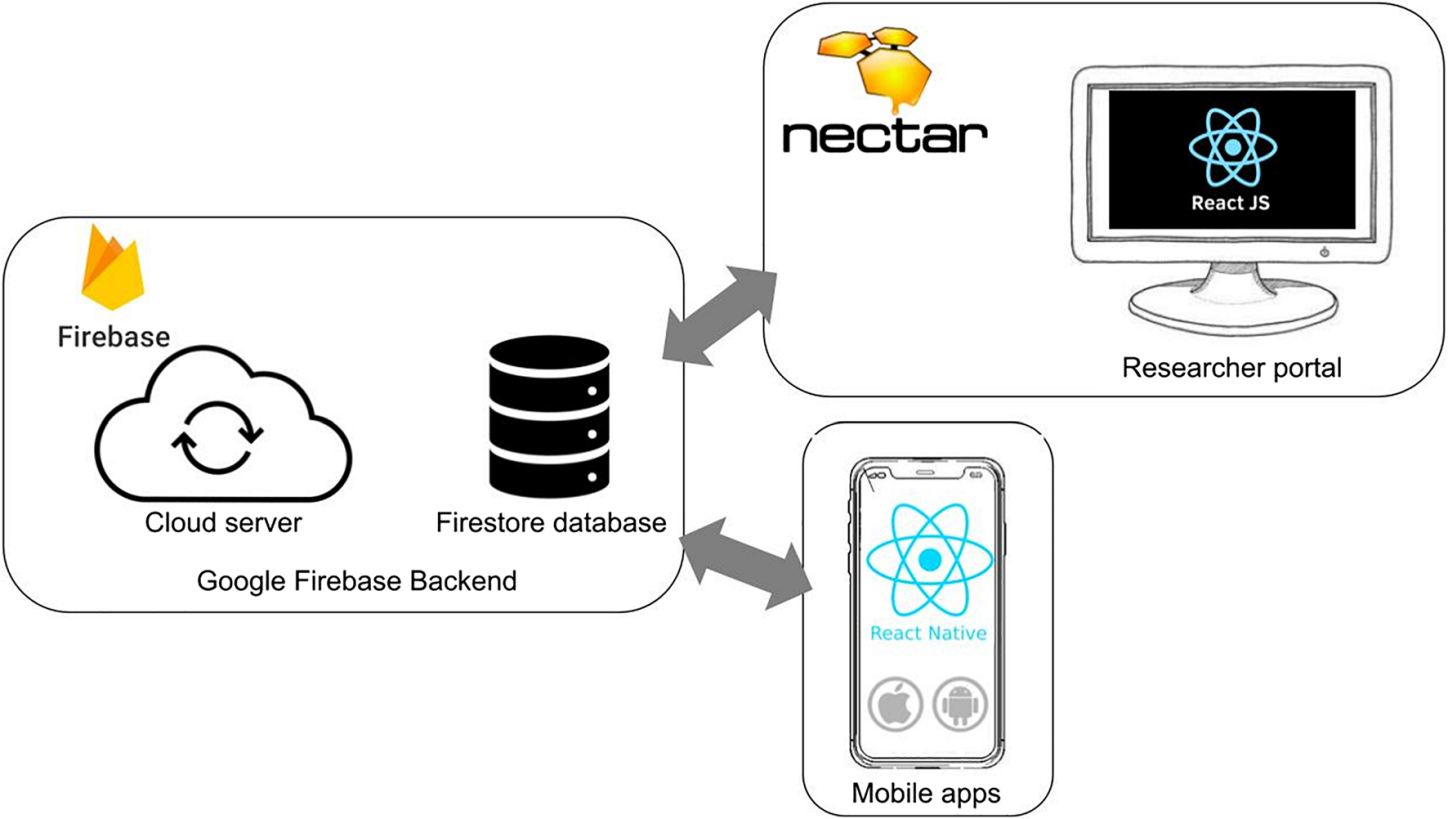
Overview of SEMA^3^ system architecture

Figure 2 provides a visual overview of the SEMA^3^ system architecture. SEMA^3^ comprises a backend hosted entirely on Google’s Firebase services; a frontend web-application (*researcher portal)* built using the Node and React frameworks, written in JavaScript and available on the National Research Cloud for Australia (NeCTAR); and mobile applications (for iOS and Android) built using React Native, written in JavaScript, Java, and Swift. The backend uses Google’s serverless “Cloud Functions”, along with “Cloud Firestore” as the main database and “Cloud Storage” for file-based storage. The researcher portal communicates with the backend via HTTPs and the Firebase JavaScript software development kit (SDK). The mobile apps communicates with the backend via HTTPs and the React Native Firebase library, which manages iOS and Android Firebase SDK, respectively.

#### Overview of SEMA^3^ researcher portal and workflow

**Fig. 3:**
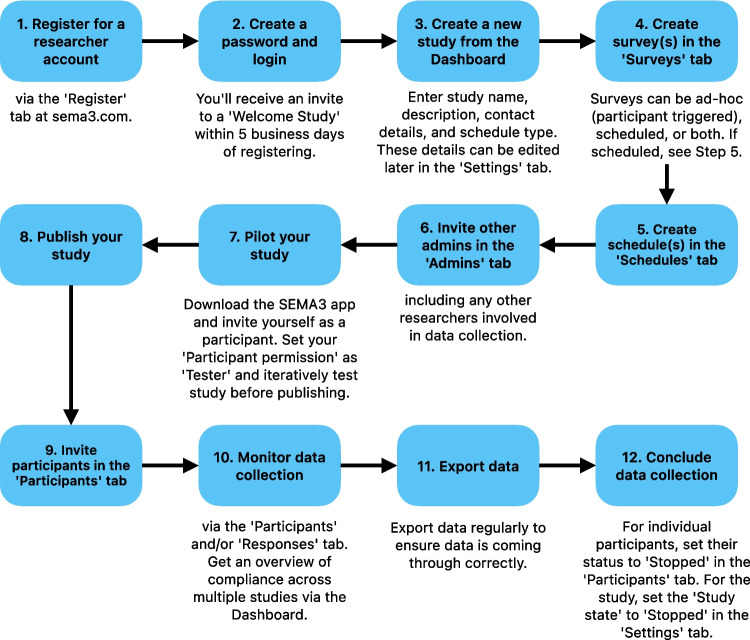
Workflow for researchers to run a study using SEMA^3^

**Fig. 4:**
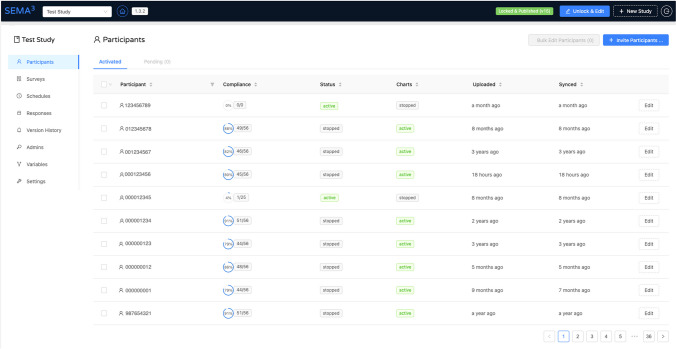
SEMA^3^ researcher portal - *Participants* tab

The aim of this section is to provide a broad overview of the key functions of SEMA^3^, from a researcher perspective.^2^ The researcher portal is hosted at https://sema3.eresearch.unimelb.edu.au and requires login credentials. Login credentials can be obtained free of charge by researchers at higher education, research, or healthcare institutions by registering at www.sema3.com/register.html. The terms and conditions for both researchers and participants are available at https://sema3.com/legal.html. Once registered, researchers can log in and see the SEMA^3^ *dashboard* (described further below). Researchers can create a new *study* from the dashboard by clicking the ‘New study’ button at the top right. Researchers will then be prompted to input basic details about the new study, including a name and brief description of the study, contact details for the responsible researcher, and a *schedule* type (discussed further below). Figure 3 illustrates the overall workflow for researchers setting up and running a study for the first time with SEMA^3^. Below we describe the main components of a SEMA^3^ study, which are also represented as tabs displayed on the left panel within a study (see Fig. 4).

### Participants

The *participants tab* within a SEMA^3^ study provides a snapshot of all participants enrolled in a given study, including their randomly generated nine-digit *participant ID*, their *compliance rate* (i.e., percentage of completed surveys), their current *status* (i.e., *active* or *stopped*), *charts status* (described further below), time of their most recent data upload, and *sync time* (see Fig. 3). Researchers can invite new participants to their study by clicking the “Invite participants” button at the top right, which will prompt researchers to enter each new participant’s name and e-mail address. Participant names are not stored in the SEMA^3^ database and e-mails are stored only in a one-way hashed format to enable verification of existing participant IDs to avoid duplication of participant profiles. This ensures participant anonymity and security of responses. Other optional fields include participant *start-date* and *end-date*, as well as a *randomization probability* (described later). Each newly invited participant is sent an invite with their participant ID to their provided e-mail address. Multiple participants can be invited concurrently either by manually adding a row for each new participant, or by uploading a .csv file containing details of multiple participants.

Once participants are invited, researchers can edit their settings (e.g., status, assigned surveys and schedules) either individually or in bulk from within the participants tab. Additional details about individual participants – e.g., charts of a participant’s responses to specific survey questions over time, next 20 scheduled surveys, participation start and end dates, and detailed compliance information – can be viewed by clicking on a specific participant’s ID.

#### Surveys

**Fig. 5:**
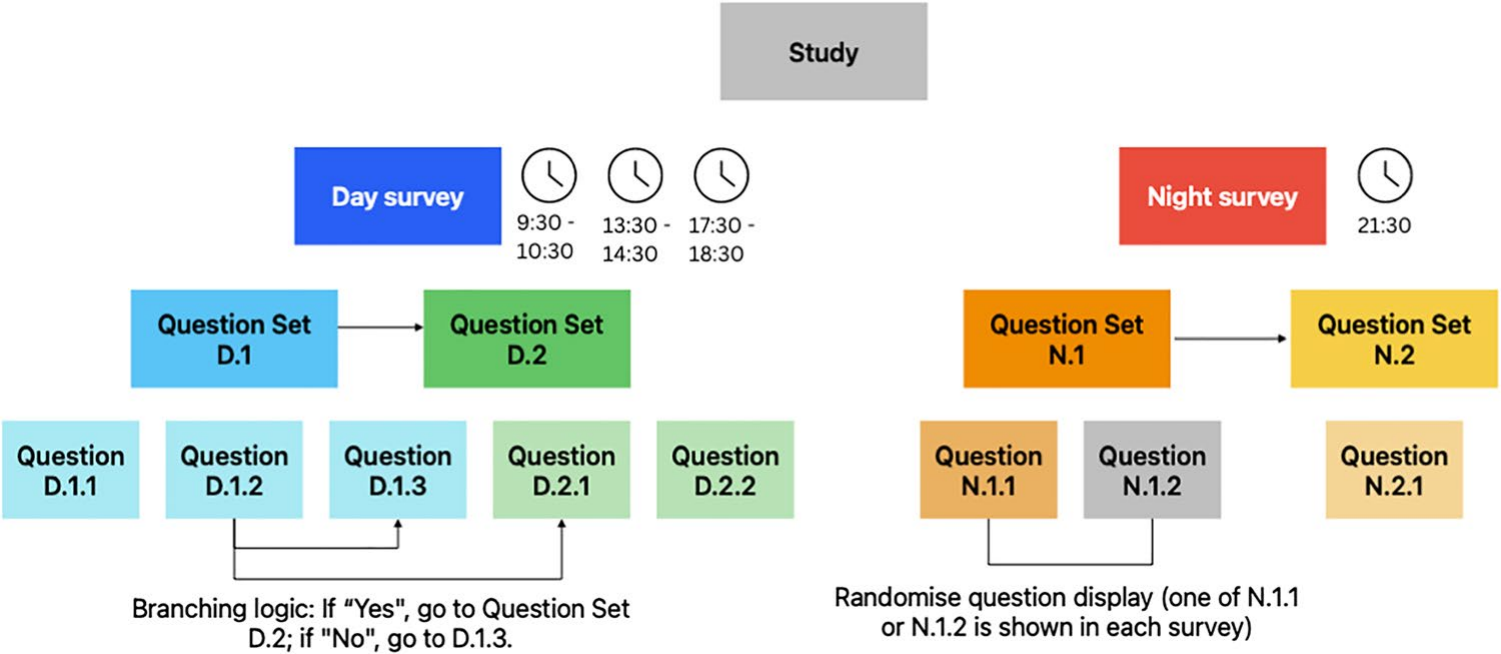
Structure of an example SEMA^3^ study

A new survey can be created in the *surveys tab.* A SEMA^3^ study comprises one or more surveys, each of which contains one or more *question sets*, which each comprise one or more *questions*. For example, as illustrated in Fig. 5, a study could contain a “day survey” with two question sets (D.1 and D.2), comprising three questions (D.1.1, D.1.2, D.1.3) and two questions (D.2.1, D.2.2), respectively; and a “night survey” with two question sets (N.1 and N.2), the first of which comprises two questions (N.1.1, N.1.2) and the second of which contains only a single question (N.2.1).

Surveys can be either *schedule-triggered* (i.e., interval- or signal-contingent sampling) or *participant-triggered* (i.e., event-contingent sampling), or both. Furthermore, the ability to include multiple surveys within a single study, each triggered by a different schedule (or participant-triggered), provides a high degree of flexibility for complex designs. Returning to the example shown in Fig. 5, the day survey could be scheduled at multiple random times throughout the day, whereas the night survey could be scheduled once each evening at a fixed time. Advanced survey flow features shown in Fig. 5 are explained below.

##### Question types

SEMA^3^ accommodates several question types: *slider questions* have customizable minimum and maximum values and labels (including image labels), can be set to hide or display the selected numeric value, and can include a researcher-determined or random initial value; *choice questions* have customizable response labels (and associated numeric values), can allow for selection of a single response or multiple responses, and response options can be presented in fixed or randomized order; *text questions* allow for free-text responses and can optionally include content validation (see below); *XY questions* allow participants to place a dot anywhere on a two-dimensional grid with customizable axes and an optional background image (e.g., to assess feelings along *arousal* and *valence* dimensions as in the Affect Grid; Russell et al., 1989); and finally *choice slider question*s are a hybrid format combining choice and slider questions types, which allow participants to select one or more items from a list and then rate each selected item along a continuous scale. Finally, instruction-only questions (requiring no response) can be included by adding a choice question without any response options.

Two additional features worth highlighting are content validation and hyperlinks. Text questions can incorporate several forms of content validation, which allow researchers to restrict responses to be entered as (a) text, (b) numbers, (c) e-mail addresses, (d) dates (dd/MMM/yyyy), or (e) times (HH:mm). For example, when asking participants what time they went to sleep, content validation ensures that participants can only input responses in a standardized time format. Finally, all question types can include clickable hyperlinks that redirect participants to a valid URL using their smartphone’s default web browser.

##### Survey flow

SEMA^3^ has several features that allow researchers to alter survey flow and question/response display order. Question sets can be displayed in a fixed or random order within a survey, and questions can be displayed in a fixed or random order within each question set. The display order of response options can also be randomized within choice and choice slider questions. Additionally, SEMA^3^ can display questions conditionally, either branching to another question set depending on an answer to a previous question, or displaying a random subset of questions within a question set. As shown in Fig. 5, conditional branching can be configured so that depending on a participant’s answer to a previous question (e.g., question D.1.2 in Fig. 5), the survey branches to a new question set (e.g., question set D.2 in Fig. 5), or the survey continues to the next question within the original set (e.g., question D.1.3 in Fig. 5). Additionally, randomizing question display within a question set can be used to show a subset of all questions within the question set (e.g., either question N.1.1 or question N.1.2 within question set N.1 in Fig. 5). The number of questions to be randomly drawn from all available questions in a set is customizable, sampling with replacement for each scheduled survey. This feature caters to *planned missingness designs* (e.g., Silvia et al., 2014).

##### Demo survey

Surveys can be tested using the demo survey feature in the SEMA^3^ app, both by researchers (*admins*) and participants of a study. Admins can demo a survey by adding themselves as a *tester* participant, which allows admins to test new surveys/questions without publishing a study – any edits to an unpublished study will only be visible to tester participants via the demo survey feature. Researchers may also find it useful to use the demo survey feature with actual study participants at the start of a study to ensure that participants understand the survey questions and know how to submit responses correctly.

### Schedules

SEMA^3^ incorporates three distinct schedule types: *weekly* (i.e., surveys scheduled from Monday to Sunday), *day index* (i.e., surveys scheduled from day 0 to day *n* of a study), and *absolute date* (i.e., surveys scheduled on specific calendar dates), however any given study is restricted to only one schedule type. Schedules are created with an intuitive point-and-click calendar style interface that researchers use to set one or more survey windows with a duration of 0 to 1439 min. Survey windows define the time-interval during which participants will receive a survey reminder notification via the SEMA^3^ smartphone app. A survey window with a duration of 0 minutes will trigger a notification at a fixed time (i.e., interval-contingent sampling), whereas survey windows with durations of 1 to 1439 min trigger notifications at random times within the specified time interval. Each survey window also has an expiry time from 1 to 1439 min, which defines the duration for which the survey remains open for completion after being triggered. For example, as shown in Fig. 5, a 09:00–10:00 survey window with a 30-min expiry implies that a survey will be delivered at a random moment between 9 a.m. and 10 a.m. and will remain open for 30 min after delivery. Thus, the earliest possible delivery time would be 9 a.m. and the latest possible delivery time would be 10 a.m., with the latest possible completion time of 10:30 a.m. Survey windows (including their expiry time portion) cannot overlap, such that in the previous example the next survey window could be scheduled to begin no sooner than 10:30 a.m.

Crucially, schedules match the time-zone of each participant’s smartphone, even if the participant is in a different time-zone to the researcher who created the schedule. This means that a survey scheduled at 10:00 a.m. will be delivered at 10:00 a.m. for a participant in Singapore, even if the researcher is in Belgium, for example.

### Responses

The responses tab provides an overview of all responses submitted by participants in a study, including what time the survey was scheduled, started, completed, and uploaded, as well as the *survey ID* (indicating which of multiple possible surveys was delivered) and participant ID associated with that response. Responses can be filtered by survey, participant, and study *version*. Admins can export data from the responses tab by clicking the “Export” button at the top-right. The filters selected in the responses tab will be reflected in the exported data. Researchers can also specify custom values to code for missing responses (by default recorded as “<no-response>”) and survey questions that were not shown, for example due to branching logic or random question sampling (by default recorded as “<not-shown>”).

#### Feedback charts

SEMA^3^ creates graphical feedback for participants in the form of *participant charts*. Participant charts provide graphs of a participant’s responses to surveys across time for slider and choice questions. These charts can be filtered by question, and responses from two questions can be overlaid in the same chart so they can be easily compared. These charts can be viewed at any time by study admins (i.e., researchers) and by participants if/when the *participant charts preview* option is set to active within the participants tab. When the participant charts preview feature is activated for a participant, they receive an e-mail containing a random alphanumeric code to access their charts securely via a web browser to maintain confidentiality of participant data. Providing participants with personalized feedback via the charts feature can serve as an incentive for participation and engagement with a SEMA^3^ study.

#### Other study components

The *version history* tab logs admin activity, including “unlocking” a study for editing and “publishing” a new version of the study. The *admins* tab provides a list of all admins (researchers) with access to a particular study, including their e-mail address and e-mail alert preferences (i.e., whether they wish to receive e-mail alerts triggered by participant compliance and latest upload time thresholds, which are set in the study *settings* tab). The *settings tab* is where information about the study in general can be edited (including *schedule type*, *study status*, *compliance alert threshold*, and *upload time alert threshold*), and where the entire study can be deleted. The *compliance alert threshold* refers to the percentage participant’s compliance must drop below for admins to be alerted (via e-mail). Similarly, the *upload time alert threshold* refers to the number of hours that must have elapsed since the participant’s last response, before admins are alerted.

#### Dashboard

**Fig. 6:**
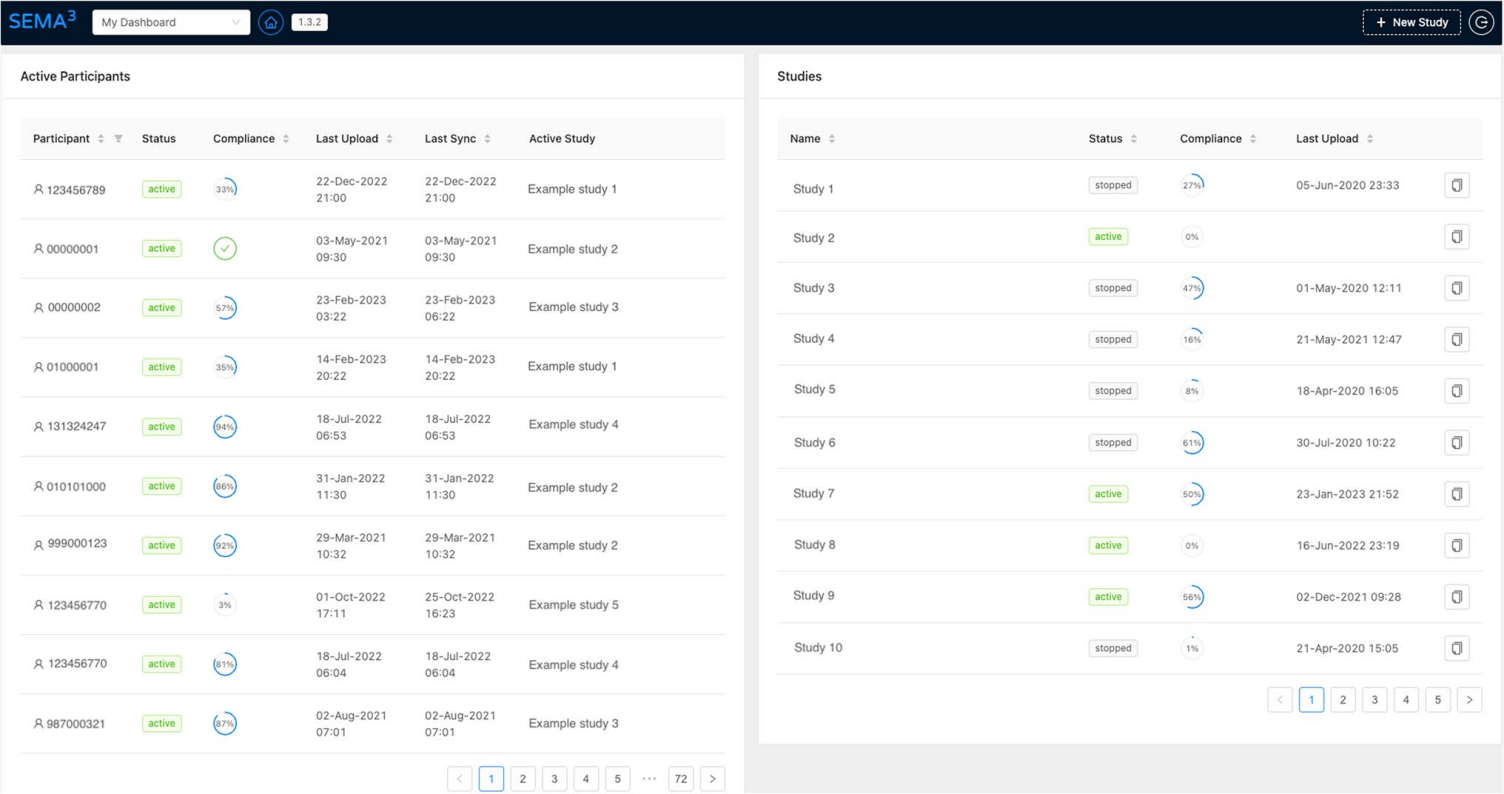
SEMA^3^ researcher portal dashboard

*My dashboard* provides an overview of all SEMA^3^ studies that an individual researcher is currently administering (see Fig. 6), including overall *compliance* (i.e., proportion of scheduled surveys completed) and recency of data upload across all participants that the researcher is responsible for (see left panel in Fig. 6). This information is also available at the study level (see right panel in Fig. 6). Studies can also be deleted from the dashboard.

## Current user-base, terms of use, and researcher support

SEMA^3^ is currently used worldwide by over 1000 researchers in more than 40 countries across Oceania (e.g., Australia, New Zealand), North America (United States, Canada), Europe (e.g., United Kingdom, Norway, Sweden, Belgium, Germany, Croatia, Greece, France, Poland, Switzerland, Portugal, Romania, Spain), and Asia/Middle East (e.g., Hong Kong, Singapore, Japan, Israel, South Korea, Philippines). SEMA^3^’s primary user base comprises researchers at universities and university-affiliated research institutes. However, SEMA^3^ is also used in teaching and research hospitals, in clinical practice, and in applied organizational settings.

### Data security and privacy

We have put in place a range of measures to protect the privacy of all SEMA^3^ users and to ensure that collected data are stored securely. We distinguish between two types of SEMA^3^ users: researchers and participants. To protect participants’ privacy, we do not store their personally identifying information (e.g., names, e-mails) in SEMA^3^. Participant on-boarding requires a valid e-mail address, which is used to send participants an invitation to a particular SEMA^3^ study (via the third-party service, MailGun). However, during on-boarding, participants also receive a unique nine-digit random numeric identifier (SEMA-ID); this is the only identifier associated with participants and their survey responses after the initial on-boarding process. SEMA-IDs are linked to hashed (encrypted) e-mails to enable one-way mapping (e-mail ➔ SEMA-ID) for the purpose of verifying whether a participant has an existing SEMA-ID when they are on-boarded into a new SEMA^3^ study. Reverse mapping (SEMA-ID ➔ e-mail) is not possible to prevent participants’ survey data from being re-identified. Participants can request their data to be deleted by contacting the researcher(s) administering their SEMA^3^ study. Finally, researchers can enable participants to access their SEMA^3^ survey responses via a graphical feedback feature, which is accessed via a secure link from within the SEMA^3^ smartphone apps and requires two-factor authentication. Researchers’ names and e-mail addresses are stored in the SEMA^3^ researcher portal to allow research teams to view and manage who has admin access to a SEMA^3^ study. This information is only visible to other researchers with admin access for that particular study.

We take great care to ensure that personal information is handled, stored, and disposed of confidentially and securely. Raw SEMA^3^ data can only be accessed by researchers with admin access to a SEMA^3^ study and, if required, by members of the SEMA^3^ development team. To access the SEMA^3^ researcher portal, researchers must hold a valid admin account and log in securely with their e-mail and password. SEMA^3^ uses Google Firebase services, including Firebase Cloud Functions, as the main API to communicate between the SEMA^3^ researcher portal, SEMA^3^ apps, and the SEMA^3^ cloud server (also hosted by Google). All communication is encrypted using industry standard (HTTPS) data security protocols. SEMA^3^ survey data are stored in an instance of Google’s cloud-based Firestore database, which encrypts data at rest and restricts access to authorized users. Google Firebase (including Firestore database) and MailGun process data on behalf of SEMA^3^ in accordance with their standard terms of service, which incorporate appropriate safeguards (including standard contractual clauses) where the data includes any personal data from the EU or UK (for details, see https://cloud.google.com/terms/data-processing-addendum; and https://www.mailgun.com/legal/dpa/). A detailed overview of our privacy policy, including details of our data storage and security measures, see https://sema3.com/legal.html#h-3.

The web application is deployed on the NeCTAR Research Cloud with the University of Melbourne availability zone. All programming and technical maintenance of SEMA^3^, including management of virtual machines, is undertaken by the Melbourne eResearch Group (MeG; www.eresearch.unimelb.edu.au) at The University of Melbourne. MeG are involved in a multitude of security-oriented research projects including large-scale biomedical projects, and projects with defense agencies, intelligence communities and industry. The SEMA^3^ platform adheres to strict access control policies with all non-essential services turned off and limited physical access. The facility is located in a secure data center at The University of Melbourne with swipe card access to a limited set of authorized individuals.

### Researcher support

Researchers have access to a comprehensive user guide and FAQ and troubleshooting document (available via https://sema3.com/manual.html). This user guide introduces research users to all SEMA^3^ functions and provides guidance to optimize study set up and participant experience, as well as minimizing the risk of avoidable issues while using the platform. We aim to continually update this document to ensure it reflects the latest available features and up-to-date advice. SEMA^3^ provides free e-mail support to researchers to assist with queries and troubleshooting. However, as a gratis research platform, we note that e-mail responses can sometimes be delayed due to limited resources and we cannot provide any minimum service guarantees.

### Limitations of SEMA^3^

As we summarized above, SEMA^3^ is a comprehensive, flexible, and highly intuitive smartphone-based daily life survey research platform. However, we also wish to acknowledge some limitations of the platform. First, there are some Android devices (e.g., Huawei, Oppo, and Realme) that are known to have compatibility issues with the SEMA^3^ Android app. To the best of our knowledge, these issues are due to manufacturer-specific variations in the Android operating system, as Android phones can be made by any manufacturer, whereas iOS devices have a single manufacturer (i.e., Apple). These (and other Android) devices may have different default settings, such as stricter low-power mode settings, that can disrupt scheduled notifications in the SEMA^3^ app. Participants with these brands of Android devices may be able to receive SEMA^3^ notifications reliably after manually changing their notification settings (see FAQ and Troubleshooting, available at https://sema3.com/manual.html), and some researchers have reported no issues with running SEMA^3^ on all brands of Android devices. We recommend carefully testing with a range of devices and, if necessary, screening participants and/or providing participants with instructions to ensure battery optimization and notification settings are unrestricted to ensure SEMA^3^ notifications are delivered reliably.

Second, participants can change the notification settings on their phones (regardless of phone type) to either stop or delay notifications across all phone types. Whilst this limitation is not unique to the SEMA^3^ app, it creates the potential for participants to miss more survey notifications than they otherwise would, therefore reducing compliance. We recommend asking participants to ensure notifications are turned on for the SEMA^3^ app for the duration of a given study.

Third, SEMA^3^ was deliberately designed to limit participation to one SEMA^3^ study at a time. If a participant is set to “active” in one SEMA^3^ study, then that participant cannot be invited to another SEMA^3^ study using the same e-mail address (and associated participant ID) with which they registered for the first study. For this reason, it is important to ensure participants are set to “stopped” once their participation in a study has concluded, so that they can be added to other studies in the future. This design feature is in place because participating in multiple experience sampling studies simultaneously can increase participant burden (Hasselhorn et al., 2022; Stone et al., 2003). When participants are completing multiple studies using the same app, their responses from one study may influence the other. In turn, data quality and quantity for both studies could be undermined, due to increased careless responses or lower compliance (Wen et al., 2017).

Finally, while SEMA^3^ is set up to retrieve time-zone information for participant responses, this information is not always available from some iOS or Android devices. For this reason, we recommend recruiting participants in no more than one or two time-zones per study and for researchers to communicate to participants the importance of notifying them of any time-zone changes during the study. This information is necessary for interpreting the survey time/date-stamps that are recorded in the exported data.

### Future of SEMA^3^

SEMA^3^ is (and will remain) available free-of-charge to eligible researchers around the world. Further, we will make a read-only version of the source code available to researchers upon request (via e-mail to the corresponding author). Finally, we intend to make SEMA^3^ source code publicly accessible and editable (i.e., open source) in the future.

We have an extensive list of future SEMA^3^ upgrades that we are gradually developing and deploying, some of which are currently undergoing Beta testing (at the time of publication). The first Beta feature is *random assignment*, which can be used for micro-randomized trials or experiments (see Neubauer et al., 2023). This feature is currently limited to randomly displaying one out of two questions within a single question set (which must contain exactly two questions). Each participant and/or occasion (i.e., survey window) can be assigned a unique probability (between 0 and 1) of receiving the first vs. second question within the to-be-randomized question set, allowing for multilevel or even cross-classified randomization.^3^ The second Beta feature is participant-specific variables. These variables can be imported via a .csv file that replaces variables in questions with unique variables for each participant. For example, if a participant is asked to provide the types of exercise they regularly engage in baseline, researchers could automatically use such exercise information in subsequent surveys to ask participants if they have engaged in those specific activities.

### Platform sustainability

We intend to continue maintaining and upgrading SEMA^3^ to ensure it remains an accessible EMA platform for years to come. SEMA^3^ has guaranteed funding to maintain current functionality for the foreseeable future. We also invite researchers with access to research funding, or who are applying for grant funding for projects using SEMA^3^, to consider making voluntary financial contributions (see https://go.unimelb.edu.au/gb7s). Up to date information about the platform and its latest features and licensing agreements can be found on the SEMA^3^ website https://sema3.com/.

## Conclusion

SEMA^3^ is a free, flexible, and user-friendly research platform for designing and administering daily life surveys on Android and iOS smartphones. Given the increasing popularity of daily life survey methods and demand for smartphone-based research platforms, there is space and need for research platforms such as SEMA^3^. We have provided an overview of basic and advanced features of SEMA^3^ that can cater to a variety of research topics across fields, and outlined the steps involved in designing a study using SEMA^3^. Our vision is that SEMA^3^ allows researchers globally to expand their methods into assessing thoughts, feelings, and behaviors in everyday life, regardless of funding availability or technical experience.

## Data Availability

Not applicable.

## Footnotes

1 As an aside, questions about the ecological validity of lab experiments have also been raised about research with rodents, whose behaviour and biology appear strikingly different in their natural environments versus in the lab (Beura et al., 2016; Brydges et al., 2011; Hylander & Repasky, 2016).

2 Although this overview of the SEMA^3^ platform was accurate at the time of publication, SEMA^3^ is continually evolving as we better understand and address researchers’ needs. An up-to-date overview of the functionalities of the platform, as well as more details of the SEMA^3^ app for participants, is available via https://sema3.com/manual.html

3 A participant probability determines how likely that participant is to be shown the first (vs. second) randomized questions across all occasions (or in the long-run). In contrast, an occasion probability determines how likely all participants in the study are to be shown the first (vs. second) randomized question at each occasion.

## Code availability

To request read-only access to the SEMA3 source code, send an e-mail to the corresponding author, Peter Koval (p.koval@unimelb.edu.au).
